# Plant–Aphid Interactions Under Elevated CO_2_: Some Cues from Aphid Feeding Behavior

**DOI:** 10.3389/fpls.2016.00502

**Published:** 2016-04-13

**Authors:** Yucheng Sun, Huijuan Guo, Feng Ge

**Affiliations:** State Key Laboratory of Integrated Management of Pest Insects and Rodents, Institute of Zoology, Chinese Academy of SciencesBeijing, China

**Keywords:** elevated CO_2_, aphid, nitrogen metabolism, plant defenses, water potential, legumes

## Abstract

Although the increasing concentration of atmospheric carbon dioxide (CO_2_) accelerates the accumulation of carbohydrates and increases the biomass and yield of C3 crop plants, it also reduces their nitrogen concentration. The consequent changes in primary and secondary metabolites affect the palatability of host plants and the feeding of herbivorous insects. Aphids are phloem feeders and are considered the only feeding guild that positively responds to elevated CO_2_. In this review, we consider how elevated CO_2_ modifies host defenses, nutrients, and water-use efficiency by altering concentrations of the phytohormones jasmonic acid, salicylic acid, ethylene, and abscisic acid. We will describe how these elevated CO_2_-induced changes in defenses, nutrients, and water statusfacilitate specific stages of aphid feeding, including penetration, phloem-feeding, and xylem absorption. We conclude that a better understanding of the effects of elevated CO_2_ on aphids and on aphid damage to crop plants will require research on the molecular aspects of the interaction between plant and aphid but also research on aphid interactions with their intra- and inter-specific competitors and with their natural enemies.

## Introduction

Since the industrial revolution, atmospheric CO_2_ concentrations have increased from 280 ppm to approximately 400 ppm due to anthropogenic effects, i.e., deforestation and fossil fuel combustion. These increases in atmospheric CO_2_ concentrations have serious implications for global warming and climate change (Stocker et al., 2013). Although changes in climate have been anticipated to greatly affect agricultural ecosystems (Fuhrer, 2003), increases in atmospheric CO_2_ concentration alone can also be very important because they can directly affect plant physiology and indirectly alter interactions between plants and herbivores and plant pathogens (Robinson et al., 2012). These altered interactions may then lead to more severe and frequent outbreaks of pest insects and plant diseases in agricultural ecosystems (Percy et al., 2002).

To understand how elevated concentrations of atmospheric CO_2_ could increase pest problems, we must first recognize that increases in CO_2_ tends to increase the growth of plants by enhancing their photosynthetic rate, resulting in higher yields for most C3 crops (Ainsworth and Rogers, 2007). Under elevated CO_2_, however, C3 crop plants exhibit decreases in nitrogen (N) and other trace elements, i.e., zinc and iron (Bloom et al., 2010). These decreases reduce the nutritional value for herbivorous insects and may therefore change their feeding behaviors (Myers et al., 2014). For those insects that chew leaves, a reduction in the N concentration in crop tissue and the resulting increase in the carbon/nitrogen ratio (C:N ratio) under elevated CO_2_ could cause these insect pests to consume more leaves to meet their N needs (Bezemer and Jones, 1998; Sun and Ge, 2011). In addition, leaves grown under elevated CO_2_ decrease their ability to produce jasmonic acid (JA), a hormone that contributes to plant defenses against chewing insects (Zavala et al., 2008).

Elevated CO_2_ may also increase the damage to crops caused by phloem-sucking insects including aphids. Aphids feed exclusively on the phloem sap and are very sensitive to changes in plant quality caused by climate change (Pritchard et al., 2007). Recent meta-analysis result shows that aphids tend to perform better under elevated CO_2_ on average (Robinson et al., 2012). The conclusions from many statsitically signficant researches, however, exhibit idiosyncratic responses of aphids in terms of population abundance, fecundity as well as survival (summarized in **Table 1**). Although predictions are difficult, it is nevertheless useful to determine why some aphids are more fit while others are less fit under elevated CO_2_. A mechanistic understanding can help make sense of these contradictory results. Previous study demonstrates that the effect of elevated CO_2_ on plant, which includes C and N assimilation, secondary metabolism, plant stomatal conductance as well as leaf temperature, could in turn affect aphid population numbers and growth (Ainsworth et al., 2006; May et al., 2013). Futhermore, the feeding behavior of aphids and their interaction with host plant under elevated CO_2_ are largely ignored but should be crucial to the understanding of idiosyncratic responses. The aim of this review is to highlight overlooked processes and new discoveries that how elevated CO_2_ affects the components of plant leaves and how these effects alter the different feeding phases of aphids. We also suggest some possible molecular mechanisms underlying the interactions between aphids and their host plants under elevated CO_2_.

**Table 1:** Potential mechanisms regarding aphid performance respond to elevated CO_2_

Potential mechanism	Aphid–host plant system	Response	Parameter	Reference
Alters absorption of foliar amino acid or	*Acyrthosiphon pisum – Medicago sativa*	Positive	Population abundance	Ryalls et al., 2015
changes the sap flow of plant	*Acyrthosiphon pisum – Medicago truncatula*	Positive	Population abundance,feeding efficiency	Guo et al., 2013
	*Aphis gossypii – Gossypium hirsutum*	Unchanged	Growth rate	Sun et al., 2009
	*Rhopalosiphum padi – Hordeum vulgare*	Positive	Population abundance, intrinsic rate of population	Ryan et al., 2015
	*Aphis fabae – Cardamine pratensis*	Positive	Population abundance	Salt et al., 1996
	*Myzus persicae – Solanum dulcamara*	Positive	Population abundance	Salt et al., 1996
	*Acyrthosiphon pisum – Medicago sativa*	Depend on plant genotypes	Population abundance	Johnson et al., 2014
Changes of nitrogen concentration or whole plant quality of host plant	*Myzus persicae – Bell pepper*	Negative	Pre-reproductive period, fecundity	Dáder et al., 2016
	*Phyllaphis fagi – Fagus sylvatica*	Negative	Fecundity, nymph weight, nymph weight	Docherty et al., 1997
	*Rhopalosiphum padi – Triticum aestivum*	Positive	Weight, relative growth rate, life span	Oehme et al., 2013
	*Myzus persicae – Brassica napus*	Negative	Weight, relative growth rate, life span	Oehme et al., 2013
	*Rhopalosiphum maidis – Hordeum vulgare*	Positive	Developmental duration, fecundity	Xie et al., 2014
Increase of photosynthesis	*Myzus persicae – four plant species (Careamine hirsute, Poa annua, Senecio vulgar, Spergula arvensis)*	Positive	Population abundance	Bezemer et al., 1998
Plant endophyte induced resistance	*Rhopalosiphum padi – Festuca arundinacea*	Negative	Population abundance, aphid density	Newman et al., 1999; Ryan et al., 2014a,b
Decrease of phytohoemone resistance	*Myzus persicae – Arabidopsis*	Positive	Population abundance	Sun et al., 2013
	*Acyrthosiphon pisum – Medicago truncatula*	Positive	Mean relative growth rate; feeding efficiency	Guo et al., 2014a,b
R-gene mediated resistance decreased	*Amphorophora idaei - Rubus idaeus*	Positive	Population abundance, adult mass	Martin and Johnson, 2011
Increase of leaf temperature	*Aphis glycines – Glycine max*	Positive	Population abundance	O’Neill et al., 2011
Decrease of stomatal aperture	*Acyrthosiphon pisum – Medicago truncatula*	Positive	Population abundance, feeding efficiency	Sun et al., 2015
Sensitivity to alarm pheromone	*Amphorophora idaei – Rubus idaeus*	Negative	Escape response to predator	Hentley et al., 2014
	*Sitobion avenae – Triticum aestivum*	Negative	Sensitivity to (E)-β-farnesene	Sun et al., 2010

## Aphid Feeding Behavior

Recent advances indicate that complex molecular interactions occur when aphids feed on plants. Unlike chewing insects that remove large pieces of plant tissues, aphids use their flexible and long stylets to obtain nutrients from the phloem sap and only inflict slight physical damage (Jaouannet et al., 2014). The specialized feeding behavior of aphids can be detected with electrical penetration graph (EPG) methods, i.e., EPG methods can be used to determine the locations and activities of aphid stylets, including pooled pathway phase activities, probing, salivation into sieve elements, passive uptake of the phloem sap, and xylem absorption (Tjallingii and Esch, 1993). Data on the initiation and duration of these feeding phases provide valuable cues regarding aphid activities and plant responses (Alvarez et al., 2006). Rather than simply withdrawing food from hosts, aphids can change their feeding location to avoid plant defenses or can secrete ‘effector’ proteins to suppress plant defenses (Hogenhout and Bos, 2011). To enhance their feeding, aphids can also alter host physiological traits, e.g., they can induce changes in host primary metabolism and in stomatal movement, and suppress the plant defenses (Giordanengo et al., 2010). Thus, a better understanding of aphid feeding behavior, its effects on hosts, and host responses is critical for understanding how elevated CO_2_ is likely to affect plant–aphid interactions.

## Aphid Probing and Penetration Stage and its Relation to Plant Resistance

### Influence of Plant Physical Barriers

Once they have arrived on a plant leaf, aphids must conquer host physical defenses including trichomes and waxes before they can insert their stylets into the host (Wang et al., 2004). Surface resistance is the first barrier of plant defense against aphid attack. The time that aphids spend between arriving on a leaf and making their first probe mainly reflects the physical barriers of the leaf surface including trichomes, repellent volatiles, and a thick or tough leaf surface (van Helden and Tjallingii, 1993). Plants can deter aphid attack by releasing secondary metabolites such as glucose esters and sesquiterpenes from glandular trichomes (Avé et al., 1987; Goffreda et al., 1989; Neal et al., 1990). Furthermore, a specifically expressed gene, *NtLTP1*, in the glandular trichomes of *Nicotiana tabacum* could enhance the plant’s defense against aphids (Choi et al., 2012). The changes in trichome density in response to CO_2_ are idiosyncratic. For example, trichome density increased in *Brassica rape* and *Medicago truncatula* (Karowe and Grubb, 2011; Guo et al., 2014a) but decreased in *Arabidopsis* and wheat under elevated CO_2_ (Masle, 2000; Bidart-Bouzat et al., 2005; Lake and Wade, 2009). In the legume *M. truncatula* under elevated CO_2_, the increased density of non-glandular and glandular trichomes caused aphids to spend more time before they made their first probe and to experience a prolonged pathway phase (Guo et al., 2014a). CO_2_ concentrations may affect trichome development by affecting the levels of gibberellic acid (GA), JA, and the microRNA molecule miR156. Elevated CO_2_ tends to increase plant GA content and decrease plant JA content (Teng et al., 2006; Zavala et al., 2008) and to decrease expression of miR156 (May et al., 2013). Additional research is needed, however, to clarify whether the effects of elevated CO_2_ on glandular trichome development and surface resistance to aphids is due to changes in GA, JA, and miR156.

### Phytohormone-Mediated Defenses

When the aphid stylet penetrates the plant epidermis and mesophyll, it forms a channel that permits the delivery of saliva into the phloem (Jaouannet et al., 2014). On the one hand, elicitors in aphid saliva could trigger the formation of reactive oxygen species (ROS), which in turn could induce plant defenses (Giordanengo et al., 2010). On the other hand, “effectors” in aphid saliva could suppress plant resistance and manipulate host cell processes to favor aphid feeding and colonization (Bos et al., 2010a,b; McLellan et al., 2013; Gimenez-Ibanez et al., 2014; King et al., 2014). Parameters of aphid feeding behavior revealed by EPG could reflect the intensity of plant resistance; these parameters include the minimum duration of pathway phase activity, the number of test probes, and the total time before phloem ingestion begins (Alvarez et al., 2006). In the *M. truncatula*–pea aphid system, elevated CO_2_ increased the number of test probes but decreased the total time before phloem ingestion began (Guo et al., 2014a). The inconsistent effects of elevated CO_2_ on aphid feeding parameters may result from the contrasting effects of elevated CO_2_ on the defense signaling pathways involving the phytohormones JA, salicylic acid (SA), and ethylene (ET) (Guo et al., 2014a). Elevated CO_2_ tends to enhance SA-dependent defense but reduce JA- and ET-dependent defenses in plants (Zavala et al., 2009; Guo et al., 2012; Sun et al., 2013). Furthermore, the enhanced SA signaling pathway under elevated CO_2_ caused aphids to spend more time before the first probe and reduced aphid fitness (Casteel et al., 2012; Guo et al., 2014a). The suppression of the JA signaling pathway under elevated CO_2_, however, reduces the time required by aphids to reach the phloem. In addition, elevated CO_2_ down-regulates the expression of the ET signaling pathway genes *ACC*, *SKL*, and *ERF* in *M. truncatula* under attack by the pea aphid system; this downregulation, decreases the accumulation of H_2_O_2_ and the activities of key enzymes related to ROS (Guo et al., 2014b).

Moreover, elevated CO_2_ potentially disrupts the homeostatic cross-talk between SA and JA/ET pathways by directly activating the *NPR1* (NONEXPRESSOR OF PATHOGENESIS-RELATED GENES1) gene (DeLucia et al., 2012; Zavala et al., 2013). *NPR1*-mediated suppression of JA signaling is regulated by glutathione biosynthesis (Spoel and Loake, 2011). Elevated CO_2_ changes the expression of genes that encode thioredoxins and glutathione S-transferase, which may activate the expression of *NPR1* (DeLucia et al., 2012). However, Sun et al. (2013) found that when the *NPR1* gene was knocked down, the JA-dependent defenses of *Arabidopsis* were not enhanced by elevated CO_2_, suggesting that the activation of *NPR1* may not explain the response of SA, JA, and ET signaling pathways to elevated CO_2_. Clearly, the upstream network regulating plant immunity against aphids is complex. The elicitors secreted from aphid salivary glands were recognized by the host co-receptor BRI-ASSOCIATED RECEPTOR KINASE 1 (*BAK1*) which subsequently phosphorylates *BOTRYTIS* INDUCED KINASE1 (*BIK1*). The *BAK1* and the *BIK1* complexes could jointly modulate the downstream phytohormone-mediated defense signaling pathway (Chaudhary et al., 2014; Lei et al., 2014; Prince et al., 2014). In addition to *BAK*/*BIK*, other kinases such as mitogen protein kinases (MAPKs) are also important for regulating plant defense responses against insect herbivores (Hettenhausen et al., 2015). A number of studies reported that MAPKs could regulate the JA, SA, and ET signaling pathways by activating WRKY genes (Zavala et al., 2013). It is still unknown whether elevated CO_2_ affects the JA- and SA-dependent signaling pathways by regulating upstream *BAK/BIK* or MAPK signaling. Thus, additional research is needed to determine how elevated CO_2_ affects these regulatory molecules in phytohormone signaling networks.

### Secondary Metabolite-Mediated Resistance

Many plant secondary metabolites may help plants resist aphid attack by negatively affecting the penetration pathway stage of aphid feeding. These secondary metabolites include alkaloids, steroids, foliar phenolic esters (rutin, cholorogenic acid, etc.), terpenoids, cyanogenic glycosides, glucosinolates, saponins, flavonoids, and pyrethrins (Sharma et al., 2000; Urbanska et al., 2002). For example, aphids that fed on high-saponin lines of alfafa required a prolonged time to penetrate the epidermis and mesophyll (pattern C wave) and showed a significant reduction in phloem sap ingestion (Goławska, 2007). Furthermore, different phenolic compounds seem to have different effects on the feeding parameters of aphids. Caffeic and gallic acids in cereals, for example, drastically shortened the probing phase of the grain aphid, whereas catechin prolonged the pathway phase and also decreased the number of probes by the grain aphid (Urbanska et al., 2002). On average, elevated CO_2_ increases the total phenolicsin plants by an average of 19%, condensed tannins by 22%, and flavonoids by 27% (Robinson et al., 2012). The excess of secondary metabolites in plants may help explain the increased epidermis and mesophyll resistance of plants during pathway and probing feeding stages of aphids under elevated CO_2_ (Guo et al., 2014a). Despite of increasing tannin content and phenolic compounds in whole host plant leaves, the bird cherry-oat aphid *Rhopalosiphum padi* performed better under elevated CO_2_ (Bezemer and Jones, 1998; Zhang et al., 2003). This result suggested that the tricky feeding strategy of the aphid allows it to avoid some potential defensive components. Thus, it is hard to predict the impact on aphid fitness only through surface or pathway effects.

Phenylalanine ammonialyase (PAL) and polyphenol oxidase (PPO) are two key enzymes involved in the synthesis of phenolic compounds that may be absorbed by the salivary sheath of the aphid stylet. The further polymerization of phenolic compounds causes browning of cells in contact with the saliva; such browning was associated with aphid probing activity during penetration of the epidermal and mesophyll tissues (Jiang and Miles, 1993; Urbanska et al., 1998; Han et al., 2009). PAL and PPO activities are changed by elevated CO_2_. For example, elevated CO_2_ tends to increase PAL activity but decrease PPO activity in *M. truncatula*. However, it is still unknown how changes in PPO and PAL activities under elevated CO_2_ affect the penetration phase of aphid feeding (Guo et al., 2014b).

### Resistance Expressed in the Phloem

After overcoming defenses associated with the plant epidermis and mesophyll, the aphid stylet may finally reach the phloem, but plants have ways to prevent or reduce the ingestion of phloem sap. Phloem sap contains carbohydrates, proteins, and amino acids that are essential for plant development (Gündüz and Douglas, 2009). If the phloem is impaired, plants could suffer loss of nutrients, disturbance of translocation, and increased vulnerability to infection by microbial pathogens (Dinant et al., 2010). Therefore, plants have evolved a range of defenses to inhibit phloem feeding by aphids (Will et al., 2013). The most common defense involves the occlusion of sieve tubes by the plugging of sieve pores (Knoblauch and van Bel, 1998). Two groups of sieve-tube occlusion mechanisms can be found in plants: callose deposition and protein plugging (e.g., Will and van Bel, 2006; Furch et al., 2007). The Ca^2+^ signaling pathway in plants plays a key role in sieve-tube occlusion during aphid penetration. When the stylet penetrates the sieve membrane, the high concentration gradient of Ca^2+^ between the apoplast and the sieve element lumen leads to an influx of Ca^2+^into the sieve element lumen, which induces occlusion (Knoblauch and van Bel, 1998). When this occurs, aphids must secret watery saliva into the phloem; the saliva contains proteins that bind Ca^2+^ and counteract sieve element occlusion. Thus, the time spent during salivary secretion into sieve elements reflects the defenses located in the phloem (Will et al., 2013).

Phloem resistance against aphids may be affected by elevated CO_2_. The key gene involved in callose biosynthesis is up-regulated in *Arabidopsis* under elevated CO_2_ (Li et al., 2008). Furthermore, cytosolic free Ca^2+^ is increased by elevated CO_2_ in *Commelina communis* (Webb et al., 1996). The increased production of callose and free Ca^2+^ in cells may cause aphids to spend more time in overcoming phloem resistance. EPG data consistently showed that elevated CO_2_ increased the time of salivary secretion into sieve elements when pea aphids fed on *M. truncatula* (Guo et al., 2013). Still, there is no direct evidence confirming that elevated CO_2_ increases the phloem resistance against pea aphids because of increases in callose deposition and in the Ca^2+^ signaling pathway.

### Aphid Phloem Ingestion and its Relation to Plant Nutrition

The efficiency with which aphids feed on phloem sap is determined by the nutritional composition of the sap (Douglas, 2003). Sucrose is the dominant organic compound in the phloem sap and is a crucial C source for aphids (Fisher and Cash-Clark, 2000; Douglas, 2003). Sucrose is the principal energy source for aphids and also provides the C skeleton for lipid, amino acid, and protein synthesis (Rhodes et al., 1996; Febvay et al., 1999). In potato, a mutation of the sucrose transporter *StSUT1* gene reduced the phloem sucrose content and simultaneously reduced the performance of the potato aphid (Pescod et al., 2007). Nevertheless, high concentrations of soluble carbohydrates in plant tissues often reduce aphid performance because they dilute other nutrients such as amino acids and proteins; as a consequence, the aphids must increase their consumption of phloem sap and excrete the excess sucrose as honeydew (Wilkinson et al., 1997). In contrast to carbohydrate-based nutrients, N nutrition is a limiting factor for aphid growth. The phloem sap ingested by aphids has a protein/carbohydrate ratio (w/w) as low as 0.1 while the leaf tissue ingested by chewing insects has a protein/carbohydrate ratio ranging from 0.8 to 1.5 (Behmer, 2008).

Increases in atmospheric CO_2_ accelerate photosynthesis and synthesize and transport of sucrose into the phloem, which dilutes the N concentration and increases the C:N ratio in the phloem of non-legumes (Barbehenn et al., 2004). The decreased nitrogen concentration of plants could prolonged the pre-reproductive period and decrease the fecundity of some aphids under elevated CO_2_ (Dáder et al., 2016). However, Sun et al. (2009) found that although amino acid relative concentration in the phloem of cotton plants was lower under elevated CO_2_ than under ambient CO_2_, higher amounts of free amino acids were found in cotton aphids fed on cotton grown in elevated CO_2_. These results suggested that cotton aphids under elevated CO_2_ will ingest increased quantities of phloem sap to satisfy their nutritional requirements. Moreover, the relative concentrations of predominantly essential amino acids in the phloem of barley are increased under elevated CO_2_ (Ryan et al., 2015). The latter result is consistent with the large increases in the levels of minor amino acids (most of which are considered essential) in tobacco seedlings under elevated CO_2_ (Geiger et al., 1998). These results suggest that although the total N concentration of plants is decreased, amino acids biosynthesis and translation in some non-legumes may increase under elevated CO_2_. Moreover, the mathematic model constructed by Newman et al. (2003) predicted that aphid populations tend to be larger under elevated CO_2_ if host plants have higher N supplementation, that the nitrogen requirement of aphids is low and that the density-dependent response is weak. Thus, a general explanation for the species-specific responses of aphids to elevated CO_2_ remains to be elucidated.

In legumes, elevated CO_2_ leads to a 38% increase in the quantity of N fixed from the atmosphere, which can compensate for decreases in plant N under elevated CO_2_ and cause the legumes to maintain a C:N ratio similar to that under ambient CO_2_ (Lam et al., 2012). When *M. truncatula* was infested by pea aphids, elevated CO_2_ significantly increased the concentration of total amino acids in leaves and of most individual amino acids in the phloem by enhancing the enzyme activities of N transamination (Guo et al., 2013). The increased amino acids, however, are mostly nonessential, and require the aphid endosymbiont *Buchnera* to convert them into essential amino acids (Nikoh et al., 2010). When the N-fixation ability was reduced by artificially induced mutation, the individual amino acid relative concentration in the phloem of *M. truncatula* was decreased such that *Buchnera* could no longer convert the nonessential amino acids into essential amino acids (Guo et al., 2013). These results with legumes suggest that elevated CO_2_ may increase the phloem feeding time of the pea aphid by altering amino acid metabolism, and that this response depends on a functional N fixation system. Responses of different cultivars, varieties, or genotypes of the same species to elevated CO_2_, however, can also vary. For example, Johnson et al. (2014) found elevated CO_2_ increased 86% and 56% essential amino acid concentrations and pea aphid colonization success on the high resistant cultivar ‘Sequel’ of *M. sativa*. However, elevated CO_2_ decreased 53% and 33% essential amino acid concentrations and aphid colonization on the moderate resistant cultivar ‘Genesis’. This result suggested some cultivars may become more or less susceptible to aphid attack under climate change conditions, an important consideration for determining future outcomes (McKenzie et al., 2013).

**FIGURE 1:**
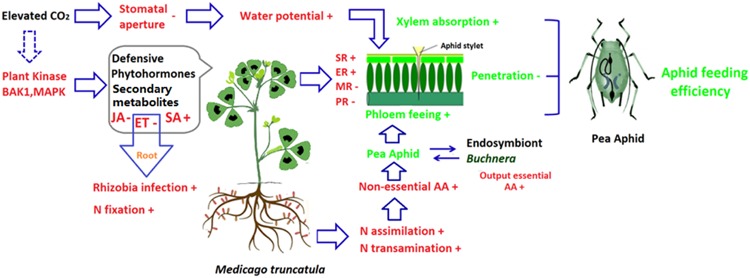
**Potential effects of elevated CO_2_ on host plant, and the cascading effects on aphid feeding using *Medicago truncatula*-pea aphid as examples.** Elevated CO_2_ affects aphid feeding efficiency in three ways. First, elevated CO_2_ modifies the phytohormone-dependent induced defenses and plant secondary metabolites derived defense. Enhancement of the salicylic acid-dependent defense pathway increased surface and epidermis resistance while the impairment of the jasmonic acid/ethylene-dependent defense pathway decreased mesophyll and phloem resistance. The changes of resistance facilitate the penetration feeding phase (the feeding phase that occurs before the stylet reaches the phloem). Second, the impairment of ethylene signaling pathway enhanced N fixation inroot, elevated CO_2_ tends to increase N assimilation and non-essential amino acid supple in the phloem. Furthermore, the aphid endosymbiont *Buchnera* could transform non-essential amino acid into essential amino acids, which increases the output of essential amino acid for aphids. Therefore, the increased amino acid supply benefits the phloem feeding of aphids. Third, elevated CO_2_ decreases stomatal conductance and transpiration, which increases the water potential in *M. truncatula*. As a result, aphid xylem feeding and osmotic pressure regulation are enhanced under elevated CO_2_. These three effects of elevated CO_2_ (alteration of host plant defenses, of amino acid supply in the phloem, and of host and aphid water status) greatly affect aphid feeding efficiency. AA, amino acid; BAK1, BRI-ASSOCIATED RECEPTOR KINASE 1; ER, epidermis resistance; ET, Ethylene; MAPK, mitogen protein kinases; JA, jasmonic acid; MR, mesophyll resistance; PR, phloem resistance; SA, Salicylic acid; SR, surface resistance; +, positively affected by elevated CO_2_; -, negatively affected by elevated CO_2_.

The ability to fix N is regulated by several hormone signaling pathways including the ET signaling pathway (Ma et al., 2002; Penmetsa et al., 2008). When the key gene *Mtskl* in the ET-perception pathway was mutated in *M. truncatula*, the nitrogenase activity was increased about two times (Penmetsa and Cook, 1997). Previous study has shown that elevated CO_2_ decreases the ET signaling pathway in *Arabidopsis* (Sun et al., 2013). The suppression of the ET signaling pathway in *M. truncatula* increased nodulation and N fixation ability, which thereby satisfied the increased demand for N by plants growing under elevated CO_2_. The down-regulation of the ET signaling pathway, however, is accompanied by decreased ET-mediated host resistance against the pea aphid (Guo et al., 2014b). This result suggested that in the *M. truncatula*–pea aphid system under elevated CO_2_, both nutritional and resistance effects would increase the fitness of the pea aphid by suppressing the ET signaling pathway (**Figure 1**).

## Aphid Xylem Absorption and its Relation to Plant Water Status

Aphids occasionally ingest xylem to increase their phloem feeding efficiency (Tjallingii and Esch, 1993; Douglas, 2006). Because the sugar-enriched phloem sap has an osmotic pressure that is as much as 4 to 5 times greater than that of the aphid’s haemolymph, continuously passive uptake of the phloem sap could result in aphid dehydration. To avoid self-dehydration and osmotic stress in the haemolymph during the phloem-feeding phase (i.e., to balance haemolymph osmolarity), aphids must consume a certain amount of xylem sap, which has a lower osmolarity than phloem sap (Pompon et al., 2011). This xylem-feeding behavior requires that the host plant has a relatively high plant water potential because the feeding is passive, i.e., fluid moves from plant to aphid because of a water potential gradient (Huberty and Denno, 2004; Daniels et al., 2009; Nalam et al., 2012). Aphids, like pathogens, can trigger stomatal closure, decrease leaf transpiration, and maintain the water content of the host plant by up-regulating the ABA signaling pathway. This manipulation of host stomata helps aphids absorb water from the xylem to neutralize phloem osmotic pressure (Sun et al., 2015).

Under elevated CO_2_, plants also exhibit reduced stomatal apertures and stomatal conductance. In FACE experiments, elevated CO_2_ has decreased stomatal conductance by an average of 22% for five functional plant groups that include 285 plant species (Ainsworth and Rogers, 2007). As noted, the decreased stomatal conductance reduces water loss from plants and increases plant water potential and water content (Wullschleger et al., 2002; Pritchard et al., 2007). Sun et al. (2015) found that the decreases in stomatal aperture and increases in plant water potential induced by elevated CO_2_ facilitated xylem feeding by aphids and thereby decreased aphid haemolymph osmolarity, which indicated a decreased cost of osmoregulation in aphids under elevated CO_2_.

## Conclusion and Perspectives

Recent studies have provided evidence that elevated CO_2_ alters plant resistance, nutritional value, and water status and that these changes affect certain feeding stages of aphids (**Figure 1**). The evidence also indicates that such changes and effects could be mediated by the phytohormones JA, SA, ET, and ABA (Guo et al., 2013, 2014a,b; Sun et al., 2015). In these and related studies, elevated CO_2_ stimulated the SA signaling pathway and thereby increased the epidermis and mesophyll resistance of plants. However, elevated CO_2_ decreased JA and ET signaling pathways, which reduced the total time required by aphids to reach the phloem. The decreased ET signaling pathway also increased the N fixation ability of legumes and thereby increased their synthesis of amino acids, which in turn increased amino acid acquisition by aphids (Guo et al., 2013). Moreover, elevated CO_2_ decreased stomatal aperture and increased plant water potential, which thereby increased aphid xylem absorption and enhanced aphid osmoregulation (Sun et al., 2015). Nevertheless, transcriptomic evidence shows that elevated CO_2_ has a wide range of effects on plant metabolism (including C and N assimilation, secondary metabolism, and transportation), all of which may affect aphid performance (Ainsworth et al., 2006; May et al., 2013). Thus, the effects of elevated CO_2_ on the interaction between plants and aphids cannot be understood by simply relating one aspect of plant quality to a specific feeding phase of the aphid. Because the responses to elevated CO_2_ differ among plant species, it is currently difficult to generalize about how further increases in concentrations in atmospheric CO_2_ affects aphid feeding and damage. An increased understanding of the molecular mechanisms underlying the recognition and interactions between plants and aphids should increase our ability to predict aphid damage under elevated CO_2_.

In addition to changes in aphid feeding behavior, changes in aphid physiology must be considered to understand how aphid performance is affected by elevated CO_2_. Some studies have reported increases in aphid growth rate and fecundity under elevated CO_2_, which suggests that elevated CO_2_ increases aphid fitness and increases the probability of aphid outbreaks. At present, we have some understanding of what happens but we do not know how it happens. Like chewing insects, aphids could sense and respond to nutritional changes in host plants by regulating a complex regulatory network involving the insulin-related peptides, the target of rapamycin (TOR), ecdysteroids, and juvenile hormone (Badisco et al., 2013). For example, TOR acts as a central regulator of protein synthesis by sensing and integrating signals from amino acid nutrition, while the insulin signaling pathway is responsible for sensing carbohydrate-derived nutrients (Grewal, 2009). Thus, research is needed on how these two nutrient-sensing and regulatory pathways in aphids affect vitellogenins and juvenile hormone/ecdysone when aphids feed on plants with increased C:N ratios under elevated CO_2_.

Herbivorous insects can be affected by environmental change via changes in host physiology and chemical composition or via changes in competitors or natural enemies (Awmack and Leather, 2002). Elevated CO_2_ affects aphid performance from the level of individual physiology or even molecular function to the level of the ecosystem (Sun and Ge, 2011). The effects of elevated CO_2_ on individual plants and aphids may differ from the effects involving the entire ecosystem and multiple trophic levels because responses to elevated CO_2_ may differ among trophic levels. It is well known that elevated CO_2_ has bottom-up effects on the feeding behavior and population size of aphids, but the situation becomes more complicated when aphid–aphid interactions or top–down effects involving natural enemies are considered. For example, aphid species, or even different genotypes within the same species, differ in their responses to elevated CO_2_ (Mondor et al., 2005), and these differences might affect the outcome of intra- or inter-specific competition between aphid species or genotypes (Stacey and Fellowes, 2002; Sun et al., 2009). Furthermore, some reports indicate that parasitoids and predators are more abundant or effective under elevated CO_2_ (Percy et al., 2002; Chen et al., 2005) and that aphids are less sensitive to alarm pheromones under elevated CO_2_ (Awmack et al., 1997; Mondor et al., 2004). It seems that enhanced top-down effects on aphids under elevated CO_2_ may strongly alter the effects of aphids on host plants (Hentley et al., 2014).

The different feeding strategies evident in aphid responses to environmental changes are possibly driven by synchronous adaptation to host and environment. Because it directly affects herbivorous only weakly, elevated CO_2_ mainly influences herbivorous insect by altering the host plant (Yin et al., 2010). Thus, understanding plant–aphid interactions is likely to be central to understanding how aphids respond to elevated CO_2_. We suggest that molecular tools be used to better understand how the host plant ‘recognizes’ the aphid and vice versa; this research might focus on salivary secretions (the most obvious ‘signal’ available), which could trigger various molecular responses in the host that then affect the aphid in various ways. Although the knowledge from literatures shows that aphids may have species-specific molecular interaction with the hosts, it is believed that the genetics and physiology governing plant–aphid interactions have many commonalities rooted in their phylogenies so that understanding the complexity of interaction will provide meaningful insights into aphids acting on different kinds of plants and aid us in using them to our best advantage. Given increasing concentrations of atmospheric CO_2_ and climate change, new crop varieties will be needed that can produce sustainable yields in spite of the changing environment and the potential for increased pressure from aphids and other pests. The development of such crop varieties will be facilitated by a better understanding of the interactions between plants and aphids at molecular, community, and ecosystem levels.

## Author Contributions

YS and HG wrote this article, FG revised it.

## Conflict of Interest Statement

The authors declare that the research was conducted in the absence of any commercial or financial relationships that could be construed as a potential conflict of interest.
